# A pragmatic application of endobronchial ultrasound-guided transbronchial needle aspiration: a single institution experience

**DOI:** 10.1186/s12890-019-0909-4

**Published:** 2019-08-20

**Authors:** Nicola Bailey, Zoe Krisnadi, Raena Kaur, Siobhain Mulrennan, Martin Phillips, Neli Slavova-Azmanova

**Affiliations:** 1 Cancer and Palliative Care Research and Evaluation Unit (CaPCREU), School of Medicine, The University of Western Australia, M581, 35 Stirling Hwy, Crawley, 6009 Australia; 2 Department of Respiratory Medicine, Sir Charles Gairdner Hospital, 1 Hospital Avenue, Nedlands, 6009 Australia; 3 School of Medicine and Pharmacology, The University of Western Australia, M507, 35 Stirling Hwy, Crawley, 6009 Australia

**Keywords:** Endobronchial ultrasound, Transbronchial needle aspiration, Lung cancer, Staging, Diagnosis

## Abstract

**Background:**

Endobronchial ultrasound-guided trans-bronchial needle aspiration (EBUS-TBNA) is minimally invasive technique used for diagnosis and/or staging of benign and malignant pulmonary and non-pulmonary disease. Previous studies have established the utility of EBUS-TBNA in narrowly defined indications and populations. In this pragmatic ‘real world’ study we have analysed the use of EBUS-TBNA for a variety of clinical presentations and its clinical application in conjunction with other invasive investigations.

**Methods:**

All EBUS-TBNA procedures performed at Sir Charles Gardiner Hospital in 2012–2014 were reviewed retrospectively, using relevant hospital databases.

**Results:**

A total of 327 patients underwent 337 EBUS-TBNA procedures. EBUS-TBNA procedures were used to diagnose a wide spectrum of benign and malignant conditions. The main application was in the diagnosis and staging of malignant conditions (70.6%), and in the diagnosis of benign conditions such as sarcoidosis 40 (12.2%), and silicoanthracosis 17 (5.2%). EBUS-TBNA was sufficient to diagnose and stage the disease as a single stand-alone invasive procedure in 191 (59.2%) patients. EBUS-TBNA was the final invasive procedure undertaken in 283 (87.6%) patients. Only 13.3% of non small cell lung cancer (NSCLC) patients who had EBUS-TBNA as a first investigation required multiple procedures compared to 51.1% of all NSCLC patients undergoing EBUS-TBNA. Overall sensitivity, specificity, NPV and diagnostic accuracy for EBUS-TBNA were 89.7, 100, 85.1 and 89.9%, respectively and three minor complications (0.9%) occurred as a result of the procedure.

**Conclusions:**

EBUS-TBNA was undertaken for a wide variety of clinical conditions. Good diagnostic accuracy and safety profiles were demonstrated for the procedure, supporting its application as a first line investigation in the diagnosis and/or staging of a range of malignant and benign conditions. Our study was unique in its documentation of the use of EBUS-TBNA in a real-world setting in conjunction with other invasive modalities. EBUS-TBNA was utilised as a stand alone invasive procedure in more than half of the patients. Importantly, in NSCLC, when EBUS-TBNA was performed as primary diagnostic and staging investigation, less patients underwent subsequent invasive procedures.

## Background

Endobronchial ultrasound-guided (EBUS) procedures utilise ultrasound imaging for visualisation and sampling of lung tissue and lymph nodes through the wall of the trachea and bronchi. EBUS is a minimally invasive procedure with a very low complication rate, which has become standard practice in many institutions internationally [1, 2]. The procedure can be used in the diagnosis of a wide range of benign and malignant conditions involving the lung parenchyma and hilar or mediastinal lymph nodes, and plays a major role in the staging of malignancies [1].

EBUS is available in two forms: EBUS guided trans-bronchial needle aspiration (TBNA) via the linear probe (EBUS-TBNA) and the EBUS radial probe via a guide sheath (EBUS-GS). Each form has specific benefits and services different patient disease presentations.

EBUS-TBNA uses a dedicated bronchoscope and allows real-time TBNA of mediastinal and hilar lymph nodes and masses. A frequent application of EBUS-TBNA is in the accurate nodal staging of lung cancer [1, 3], however the procedure is also utilised for assessment of mediastinal/hilar lymphadenopathy in patients with other malignancies [4, 5], and for the pathological diagnosis of enlarged lymph nodes part of lymphoproliferative [6], infectious [5, 7] and benign diseases [8, 9]. The technique also enables tissue sampling of mediastinal lesions [10] and parenchymal lung lesions located adjacent to the central airways [11, 12]. EBUS-TBNA sampling provides sufficient material for molecular biomarker testing of clinically significant mutations allowing for selection of candidates who will respond to specific targeted treatment regimens [13]. In staging lung cancer, EBUS guided TBNA has been established as superior to conventional bronchoscopy and TBNA [14]. EBUS-TBNA also has the advantage of providing both diagnosis and staging, and is now recommended by Australian and International guidelines as the initial diagnostic intervention in appropriate patients over the previous gold standard mediastinoscopy [15–18].

Computed tomography guided transthoracic needle aspiration (CT-TTNA) can be used to gain peripheral tissue samples from the lung and provides good diagnostic accuracy, however carries a risk of pneumothorax [19] and exposes the patient to radiation.

A strong evidence base addressing the effectiveness, efficacy and safety of the EBUS-TBNA technique already stands, however most studies have analysed the diagnostic performance in narrowly defined populations [8, 16, 20]. Furthermore, no studies have reviewed the use of EBUS in conjunction with other invasive techniques including their temporal relationship in the diagnosis and staging process. The focus of this pragmatic study was to examine the utility of EBUS-TBNA in a clinical setting, free from the constraints of prespecified populations, and its use in conjunction with other invasive tissue sampling techniques at our institution.

## Methods

### Study design and patients

We retrospectively reviewed all EBUS-TBNA procedures performed at Sir Charles Gardiner Hospital (SCGH) between 1 January 2012 and 31 December 2014. Data regarding the clinical application, diagnostic performance and safety of EBUS was extracted using relevant hospital databases. Patient details such as age, gender, smoking history and Eastern Co-operative Oncology Group Performance Status (ECOG) [21] were recorded. Other invasive and non-invasive investigations relevant to diagnosis and staging were recorded if they fell within the same provision of service as EBUS. The number and details of complications resulting from all procedures were recorded. Presence of a complication was based on post-procedural chest x-ray records and clinical notes.

Ethics approval was granted by the SCGH Human Research Ethics Committee (Ref. No 2013–233). Waiver of consent was granted for inclusion of patients who had EBUS in 2012, 2013 and January–February 2014, while patients included in the study after February 2014 provided consent for access to their medical records.

### EBUS procedures

Patients at SCGH were generally presented to the lung multidisciplinary meeting (MDM) after an initial CT scan of the thorax and upper abdomen and, in the majority of the cases results of a positrom emission tomography (PET) scan, which guided recommendations for an EBUS-TBNA investigation. Mediastinal lymph nodes were sampled if they were enlarged radiologically (> 1 cm) in the short axis and/or were PET positive. In general, if there was a pulmonary mass or nodule and evidence of mediastinal or hilar lymph node involvement, the lymph node was sampled first on the grounds that diagnosis and staging might be achieved with one procedure. Lymph node sampling was undertaken by first needling the lymph node that constituted the highest stage (N3) and progressing down to the lowest stage. Rapid on-site evaluation (ROSE) was undertaken and the adequacy of the sample was assessed by the presence of lymphocytes and evaluation by the pathologist. The site and number of lymph node stations sampled and the number of passes per lymph node were determined by the operator. At least three needle passes were made per lymph node unless the diagnostic material was reported adequate on ROSE. If the lymph node samples were non diagnostic then the pulmonary lesion would be targeted for diagnostic purposes by EBUS-GS if suitable, or otherwise by CT-TTNA. Some patients were referred to our service from other sources and may have had a diagnosis already established but required staging or sampling for molecular analysis.

Hilar and mediastinal lymphadenopathy or mediastinal masses were directly sampled by EBUS-TBNA. EBUS-TBNA was performed under general anaesthesia.

### Study definitions

For the purpose of this study, cases referred for EBUS-TBNA were divided into two categories based on the intention of the procedure: 1) to confirm, stage or exclude malignancy, 2) to obtain tissue diagnosis for suspected benign conditions, based on clinical or radiological evidence. Cases were also classified into four sub-groups, based on radiological manifestations: those with a lung mass or nodule only, those with a lung mass or nodule and hilar or mediastinal lymphadenopathy, those with a hilar or mediastinal mass only, or those with hilar or mediastinal lymphadenopathy only.

Pathology results were reported as *malignant* when malignant cells were present, and *benign* when normal lung or lymphatic tissue with no malignant cells were seen. Inadequate samples were excluded from sensitivity and specificity analysis, but included in diagnostic accuracy. Standard definitions of sensitivity and specificity were used.

Cyto- and histo-pathological specimens for cases in category one were considered true positive if malignant cells were reported and true negative if an adequate sample showed no malignant cells and further clinical follow-up, pathological findings, or medical imaging confirmed the result. False negative specimens failed to detect malignancy in an adequate sample and malignancy was later clinically, radiologically or pathologically detected in the same lymph node, mass or region.

Cyto- and histo-pathological specimens in category two were considered true positive if the sample was adequate for diagnosis of a benign condition, and false negative when the sample was normal and diagnosis was established by other means. Specimens were considered true negative if pathology was not identified in an adequate sample.

All available clinicial documentation, radiological investigations, invasive investigations and pathological results relevant to the same provision of care were reviewed and recorded. All additional invasive investigations and their temporal relationship to EBUS were recorded if they related to the same provision of service. Four patients, referred from external sources, with incomplete records were excluded from the analysis of additional invasive procedures. The minimum follow up time for records after EBUS-TBNA procedure was 12 months, however patients without a clear final diagnosis or with non-diagnostic results of EBUS were followed up for two years.

### Statistical methods of analysis

Data was analysed using the IBM SPSS Version 22 statistical software package. A descriptive analysis was performed where categorical variables were expressed as absolute and relative frequencies, and continuous variables were calculated as means.

## Results

The demographic characteristics of all patients are recorded in Table 1. The mean patient age was 63 years (age range, 18–88 years), 192 (58.7%) were male and 225 patients (68.8%) were current or previous smokers.

**Table 1:** Patient characteristics

	mean (SD)
Age at investigation (years)	63 (13)
	*n* (%)
Gender	
Male	192 (58.7)
Female	135 (41.3)
Smoker	
Current	90 (27.5)
Ceased	135 (41.3)
Never	60 (18.4)
Unknown	42 (12.8)
ECOG-PS	
0	195 (59.7)
1	111 (33.9)
2	16 (4.9)
3	4 (1.2)
4	1 (0.3)

### Indications for EBUS-TBNA and sample sites

A total of 327 patients underwent 337 EBUS-TBNA procedures. Of these, 317 patients had one and ten patients had two EBUS-TBNA procedures.

EBUS-TBNA sample site locations (reported in Table 2) included mostly lymph node targets, however some mediastinal, hilar and lung masses were also sampled. The Subcarinal station (7) had the highest frequency of sampling, being assessed in 177 (52.5%) EBUS-TBNA procedures; followed by 4R - 155 (46.0%), 11R - 94 (27.9%), 4 L - 44 (13.1%) and 11 L - 38 (11.3%).

**Table 2:** Sample sites of EBUS-TBNA

Sample site	*n* (%)
Lymph nodes	562 (93.6)
Hilar/Mediastinal Masses	37 (6.2)
Lung Mass	1(0.2)
Total	600 (100)

Of the 337 EBUS-TBNA procedures, 307 were requested for patients with suspected malignant conditions (category 1), and 30 for patients with suspected benign conditions (category 2). Within category 1, 249 (81.1%) procedures were performed for diagnosis with or without staging, 53 (17.3%) were for cancer staging, and 5 (1.6%) for material for molecular tests.

There were 297 (90.8%) category 1 patients where EBUS-TBNA was requested to confirm, stage or exclude malignancy. Of these patients 166 (55.9%) presented with a lung mass/or nodule and hilar/mediastinal lymphadenopathy, 105 (35.4%) with hilar/mediastinal lymphadenopathy, 26 (8.8%) with a hilar or mediastinal mass.

There were 30 (9.2%) category 2 patients where EBUS-TBNA was requested to obtain tissue diagnosis for suspected benign conditions.

### Further investigations

Of the 323 patients with records of their invasive investigations, EBUS-TBNA was sufficient to diagnose and stage the disease as a single stand-alone invasive procedure in 191 (59.1%) patients. One hundred and eight (33.0%) patients underwent other invasive investigations in addition to EBUS-TBNA, and 24 (6.7%) had multiple EBUS procedures (Table 3). EBUS-TBNA was the final invasive procedure undertaken in 283 (87.6%) patients. Reasons for additional invasive tests are presented in Table 4.

**Table 3:** EBUS-TBNA and other invasive investigations performed

Invasive Investigations	Lung mass or nodule and lymphadenopathy	Hilar/ mediastinal mass	Hilar/ mediastinal lymphadenopathy	Total
	*n* (%)	*n* (%)	*n* (%)	*n* (%)
Suspected malignant condition	*N* = 164	*N* = 25	*N* = 104	*N* = 293
One EBUS only	78 (47.6)	15 (60.0)	73 (70.2)	166 (56.7)
EBUS and other investigations	86 (52.4)	10 (40.0)	31 (29.8)	127 (43.3)
Suspected benign condition	*N* = 3	*N* = 1	*N* = 26	*N* = 30
One EBUS only	0 (0)	1 (100)	24 (92.3)	25 (83.3)
EBUS and other investigations	3 (100)	0 (0)	2 (7.7)	5 (16.7)
All participants	*N* = 167	*N* = 26	*N* = 130	*N* = 323
One EBUS only	78 (46.7)	16 (61.6)	97 (74.6)	191 (59.2)
One EBUS and other investigations	69 (41.3)	9 (34.6)	30 (23.1)	108 (33.4)
EBUS undertaken as first Investigation	14 (8.4)	2 (7.7)	8 (6.2)	24 (7.4)
EBUS undertaken between other investigations	6 (3.6)	0 (0.0)	3 (2.3)	9 (2.8)
EBUS undertaken as last investigation	49 (29.3)	7 (26.9)	19 (14.6)	75 (23.2)
More than one EBUS only	8 (4.7)	1 (3.8)	2 (1.5)	11 (3.4)
More than one EBUS and other invasive investigations	12 (7.3)	0 (0.0)	1 (0.8)	13 (4.0)
Confirmed NSCLC	110	11	12	133
One EBUS only	50 (45.5)	6 (54.5)	9 (75.0)	65 (48.9)
EBUS and other investigations	60 (54.5)	5 (45.5)	3 (25.0)	68 (51.1)
Confirmed sarcoidosis	1	0	39	40
One EBUS only	0 (0)	0	28 (71.8)	28 (70.0)
EBUS and other investigations	1 (100)	0	11 (28.2)	12 (30.0)

**Table 4:** Reasons for additional invasive investigations

Reason for additional invasive tests	Lung mass or nodule and lymphadenopathy	Hilar/ mediastinal mass	Hilar/ mediastinal lymphadenopathy	Total
	*N* = 89	*N* = 10	*N* = 33	*N* = 132
	*n* (%)	*n* (%)	*n* (%)	*n* (%)
Material for further tests or staging when diagnosis already achieved	58 (65.2)	6 (60.0)	12 (36.4)	76 (57.6)
High clinical suspicion of benign or malignant disease, multiple attempts for tissue confirmation	22 (24.7)	4 (40.0)	15 (45.4)	41 (31.1)
Inadequate prior sample/s	7 (7.9)	0 (0.0)	6 (18.2)	13 (9.8)
Concurrent EBUS-GS and EBUS-TBNA to ensure sufficient sample	2 (2.2)	0 (0.0)	0 (0.0)	2 (1.5)

Of the 293 patients in category 1, 166 (56.7%) required EBUS-TBNA alone (Table 3). The other 127 patients required more than one invasive investigation; 86 (52.4%) of these presented with a lung mass or nodule and mediastinal lymphadenopathy.

A single EBUS-TBNA investigation was adequate in 74.6% of cases with hilar or mediastinal lymphadenopathy on radiological studies, 61.6% with hilar or mediastinal masses and in 46.7% with lung mass or nodule and lymphadenopathy (Table 3).

### Final diagnosis of patients refered for EBUS-TBNA

EBUS-TBNA returned adequate tissue for cytopathological analysis in 96.1% of procedures. Final diagnoses are recorded in Table 5. Three-hundred and twenty four patients had definitive final diagnosis established and three patients showed resolution of an unknown benign condition. EBUS-TBNA procedures were used to diagnose a wide spectrum of benign and malignant conditions. The main application was in the diagnosis and staging of malignant conditions (70.6%), and in the diagnosis of benign conditions such as sarcoidosis 40 (12.2%), and silicoanthracosis 17 (5.2%). One patient was diagnosed simultaneously with sarcoidosis and chronic lymphocytic leukaemia.

**Table 5:** Final diagnosis of all patients *n* = 327

Final Diagnosis	All EBUS cases Number (%)
ESTABLISHED FINAL DIAGNOSIS	324 (99.1)
Malignant conditions	231 (70.6)
Primary lung malignancy	168 (51.4)
NSCLC	136 (41.6)
SCLC	27 (8.3)
Carcinoid	5 (1.5)
Recurrent lung cancer	3 (0.9)
Haematological malignancy	12 (3.6)
Other Malignancy	48 (14.7)
Benign conditions	19 (5.8)
Reactive lymphadenopathy	17 (5.2)
Bronchiogenic cyst	1 (0.3)
Paraoesophageal cyst	1 (0.3)
Infective conditions	11 (3.4)
Lung infection	4 (1.2)
Tuberculosis	6 (1.9)
Lung abscess	1 (0.3)
Other conditions	63 (19.3)
Sarcoidosis	40 (12.2)
Silicoanthracosis	17 (5.3)
Radiation pneumonitis	1 (0.3)
Castleman disease	1 (0.3)
Foregut Cyst	1 (0.3)
Amyloidosis	1 (0.3)
Berilliosis	1 (0.3)
Sarcoidosis and CLL	1 (0.3)
UNESTABLISHED BENIGN CONDITIONS	3 (0.9)

*NSCLC* Non-small cell lung cancer, *SCLC* Small cell lung cancer, *CLL* Chronic lymphocytic leukaemia

### Diagnostic performances of EBUS-TBNA

The diagnostic performance of EBUS-TBNA are recorded in Table 6. We did not identify false-positive results. The overall sensitivity, specificity, negative predictive value (NPV), and diagnostic accuracy for EBUS-TBNA were 89.7, 100, 85.1 and 89.9%, respectively.

**Table 6:** Diagnostic performance of EBUS-TBNA

	Sensitivity %	Specificity %	PPV %	NPV %	Accuracy %
EBUS-TBNA					
Overall *n* = 337	89.7	100.0	100.0	85.1	89.9
Indications for EBUS-TBNA					
Lung lesion and lymphadenopathy *n* = 175	88.0	100.0	100.0	82.2	88.6
Hilar/mediastinal mass *n* = 28	95.7	100.0	100.0	80.0	92.9
Hilar/mediastinal lymphadenopathy *n* = 134	90.4	100.0	100.0	88.9	91.0
Final diagnosis					
Malignant conditions *n* = 238	91.0	100.0	100.0	76.8	89.9
Benign conditions *n* = 99	81.5	100.0	100.0	93.1	89.9
NSCLC *n* = 142	90.8	100.0	100.0	82.0	91.5
Sarcoidosis *n* = 42	80.0	100.0	100.0	83.3	85.7

### Molecular tests

Molecular testing was predominantly undertaken in patients with adenocarcinoma for epidermal growth factor receptor (EGFR) mutation, although in the initial stages of this study such testing was undertaken after discussion at the MDM since the test did not have Government remuneration. Kirsten rat sarcoma (KRAS) mutation was not performed if the EGFR was positive, since EGFR and KRAS are mutually exclusive in a patient, and since KRAS is not yet a targetable lesion. Anaplastic lymphoma kinase (ALK) gene rearrangement testing was performed in patients with adenocarcinoma who were EGFR negative; BRAF was performed in patients with melanoma, and C-MYC mutations in patients with lymphoma. Of 60 EBUS-TBNA samples sent for molecular testing, positive results were reported for 25 (41.7%) and 35 (58.3%) were negative. KRAS mutations, which were not routinely tested, were identified in 12 (20%) samples, EGFR mutations in five (8.3%), and ALK rearrangement was demonstrated in two samples (3.3%). A BRAF gene mutation was detected in five (8.3%) patients with metastatic melanoma and a C-MYC mutation was detected in one (1.7%) patient with non-Hodgkin lymphoma.

### EBUS-TBNA complications

Three complications (0.9%) occurred as a result of EBUS-TBNA. Two procedures were complicated by haemorrhage. One case was registered on a postprocedural X-ray and resolved without additional interventions, the second occured at the time of TBNA and while haemostasis was restored during the procedure, the presence of hypoxia required an overnight admission for observation. One patient developed wheeze, dyspnoea and cough following EBUS-TBNA and was admitted for treatment and observation. There were no obvious complications during the procedure and the post-procedural symptoms were thought to be due to gastro-oesophageal reflux and laryngospasm*.*

## Discussion

This study showed that EBUS-TBNA was utilised for a wide variety of clinical conditions in our institution. The main application was for diagnosis and staging of lung cancers, with 81% of these being non small cell lung cancer (NSCLC). EBUS-TBNA has previously been shown to have an essential role in the diagnosis and staging of lung cancers. It is recommended in current international and Australian guidelines as a first line investigation of lung malignancies with mediastinal lymph node involvement [17, 18, 22], being equally as accurate and safer than mediastinoscopy [23]. Sarcoidosis was the most common benign condition diagnosed with EBUS-TBNA. The use of EBUS is expanding and it has been shown to be a valuable diagnostic tool for sarcoidosis [24].

In our study, five EBUS-TBNA procedures were indicated for the specific purpose of determining molecular characteristics of lung cancers, and molecular testing was performed in 17.8% of EBUS-TBNA procedures. With further development of targeted oncological therapies it is expected that the use of EBUS-TBNA in this area is likely to expand in the future [25].

The literature reports typical diagnostic yields for EBUS-TBNA ranging between 80 and 100% [26–29]. Our study showed similar results with accuracy of 89.9% keeping with international reports. Variation in yield between different studies can be explained by differences in hospital volume of cases, operator skills, size and number of lymph nodes sampled [30].

The relatively high diagnostic accuracy of EBUS-TBNA in our study could be due to the availability of ROSE, which allows for confirmation of true positive results, reducing the need for unnecessary additional passes, and confirmation of sample adequacy through the presence of lymphocytes [1]. Furthermore, EBUS-TBNA was performed under general anaesthetic, however there are controversial reports regarding the role of deep sedation on EBUS performance. Yarmish et al. showed higher diagnostic yeld in the deep sedation group when compared to moderate sedation in a retrospective study [31]. A systematic review of five studies including that of Yarmish et al. concluded that moderate and deep sedation used for EBUS-TBNA procedures have comparable diagnostic yields [32].

EBUS-TBNA is often used in the diagnosis of sarcoidosis with various levels of diagnostic accuracy reported. Two meta-analyses reported a pooled diagnostic accuracy of 79% (range of 54 to 93% and SD 24%) [33, 34], and the paper from Trisolini et al. showed a sensitivity of 84% (95 CI:79–88%) [34]. The EBUS-TBNA in our study demonstrated diagnostic yield for sarcoidosis of 85.7% and a sensitivity of 80%, which is in agreement with the current literature [33].

The highest observed sensitivity was for samples with malignant final diagnosis (91%), however the NPV of this group was much lower than that of cases with benign final diagnosis (76.8 and 93.1%, respectively). The low NPV mandates further sampling if clinical suspicion of alternative disease is ongoing and a negative result is obtained. In our study the sensitivity and specificity of EBUS-TBNA for NSCLC was 90.8 and 100%, respectively, higher than the median sensitivity of 89% (range 46 to 97%) reported in a recent meta-analysis [22].

This study was unique in its recognition of the clinician’s real-world use of EBUS-TBNA in combination with other invasive investigations. In the past decade, the refinement of EBUS procedures has allowed patients to experience a less invasive and expediated process in diagnosis and staging of cancer. Ideally, the initial invasive investigation for lung cancer should simultaneously provide tissue for histopathological diagnosis, allow for additional molecular testing and accurately stage the disease. Minimising procedures required to diagnose and stage malignancy spares patients additional risks associated with procedures and importantly, expediates decision making and time to treatment commencement.

In suspected cancer cases included in our study, 56.7% of EBUS-TBNA procedures were adequate as a stand alone invasive procedure for diagnosis and staging. This figure is reassuring and suggests an appropriate use of EBUS-TBNA in line with clinical practice guidelines.

Although ideally diagnosis and staging are performed simultaneously with EBUS-TBNA, our study included a wider variety of clinical presentations including patients with extrathoracic disease requiring additional staging with EBUS-TBNA or metastatic spread from an intrathoracic origin requiring other invasive modalities. Inadequate prior samples, which may represent poor modality choice (EBUS or other) was recorded for 13 patients, representing 9.8% of those who underwent tests in addition to EBUS-TBNA.

The largest radiological group requiring additional invasive sampling techniques were those with lung lesion and lymphadenopathy, where 89 (53.3%) required tests in addition to EBUS-TBNA. This group included 58 (65.2%) patients with established diagnosis where tissue sample was required for further tests or staging. Forty eight of these patients had undergone initial sampling with CT FNA, bronchoscopy, or other thechniques and EBUS was the last invasive modality. For 34 NSCLC cases EBUS-TBNA was done for staging and if it was considered as the first invasive investigation potentially multiple procedures may have been avoided. Furthermore, our study supports previous findings that when EBUS-TBNA is performed as primary diagnostic and staging method in NSCLC, patients are less likely to require subsequent invasive sampling procedures [35]. Only 13.3% of the patients who had EBUS-TBNA as a first investigation required multiple procedures compared to 51.1% of all NSCLC patients undergoing EBUS-TBNA.

Our hospital was the only institution performing EBUS-TBNA in the state at the time of data collection and thus some patients in this group may have not had access to the procedure, accounting for the choice of alternative initial invasive investigations.

The literature reports an excellent safety profile, with a complication rate of 0.15% for EBUS-TBNA [1]. In our study, two patients experienced minor haemorrhage during EBUS-TBNA procedures, however this was self-limiting requiring no intervention. One patient did require hospitalisation after laryngospasm and dyspnoea following an EBUS-TBNA procedure, however this seems to be reflective of the patient’s underlying lung disease and less likely to be secondary to the procedure itself. Our previous results showed an excellent safety profile of EBUS-TBNA in lung cancer patients [36].

We have assessed a heterogenous group of patients referred for EBUS-TBNA for different indications to one of the largest tertiary hospitals in Western Australia, and the only hospital in the state offering EBUS at the time. We recognise that this study has limitations. This was a single-centre retrospective study and the data is limited to the information available from hospital records. Due to its pragmatic approach, the study depicts the application of EBUS-TBNA procedures at our institution and our findings may have limited generalisability particularly given the performance of EBUS procedures under general anaesthesia.

## Conclusions

The diagnostic power and excellent safety profile of EBUS-TBNA already documented in the literature was reflected at our institution supporting its use as a first line investigation in patients with mediastinal and hilar lymphadenopathy with or without suspicious pulmonary nodules or masses. The application of EBUS-TBNA at SCGH is largely in the diagnosis and staging of lung cancer, with promising results to support its use in the diagnosis of a variety of benign and malignant conditions. EBUS-TBNA was used in conjunction with other invasive investigations with the aims of maximising diagnostic ability, providing material for molecular testing and accurately staging malignancy, while minimising invasive procedures required. EBUS-TBNA was effectively utilised as a stand alone invasive procedure in more than half of patients at our institution. Importantly, in NSCLC, when EBUS-TBNA was performed as primary diagnostic and staging investigation, patients underwent less subsequent invasive procedures.

## Data Availability

The datasets generated and/or analysed during the current study are not publicly available due to practical reasons but are available from the corresponding author on reasonable request.

## Abbreviations

ALK: Anaplastic lymphoma kinase
CLL: Chronic lymphocytic leukaemia
CT-TTNA: Computed tomography guided transthoracic needle aspiration
SCGH: Sir Charles Garfiner Hospital
EBUS: Endobronchial ultrasound
ECOG: Eastern Co-operative Oncology Group Performance Status
EGFR: Epidermal growth factor receptor
KRAS: Kirsten rat sarcoma
NSCLC: Non-small cell lung cancer
NPV: Negative predictive value
PPV: Positive predictive value
ROSE: Rapid on-site cytology examination
SCLC: Small cell lung cancer
TBNA: Trans-bronchial needle aspiration
